# Is uncertainty in complex disease epidemiology resolvable?

**DOI:** 10.1186/s12982-015-0028-5

**Published:** 2015-05-09

**Authors:** Wasim Maziak

**Affiliations:** Department of Epidemiology, Florida International University, 11200 SW 8th St, Miami, FL 33139 USA

**Keywords:** Epidemiology, Complex disease, Small risk, Chronic disease, Complexity, Measurement, Uncertainty

## Abstract

The imposed limitations on what we can know about nature have been long recognized. Yet in the field of epidemiology a futile search for lifestyle-related risk factors for common chronic diseases continues unabated. This has led to the production of a growing body of evidence about potential lifestyle risk factors that tend to be marginal, contradictory, irreproducible, or hard to interpret. While epidemiologists are calling for a more refined methodology, I argue that our limitation in studying complex diseases is insurmountable. This is because the study of lifestyle-related small risks requires accurate measurement of multiple behaviors-exposures over a long period of time. It is also because in complex systems such as population’s health, the effect of rich interactions between its parts cannot be predicted based on traditional causal models of epidemiology. Within complex systems, understanding the interactions between system components can be more important than the contribution of each to disease risk.

## Background

In quantum physics, mathematics, and the science of complexity, scientists have long known the limitation to what can we learn by observing and probing natural phenomena. Whether this uncertainty is an inherent property of nature or a human limitation has little bearing on the fact that some aspects of life, it seems, can never be known for sure. This is mostly due to our limited ability to measure their attributes with adequate certainty, or to predict complex interactions involved in their occurrence [1,2]. Epidemiology, as the science of determinants of population health is likely to be facing such uncertainty, especially in its quest to uncover lifestyle risks of complex diseases.

We are confronted regularly with new results of epidemiological studies about lifestyle risks to common chronic ailments such as cardiovascular, cancer, psychiatric and neurodegenerative diseases. Most of these risks tend to be marginal, contradictory, irreproducible, or hard to interpret [3]. For example, try to search the literature to know whether wine, fish, coffee, salt, fat, red meat, or more specifically certain vitamins, nutrients or anti-oxidants are good or bad for you. For you as a whole human being and over your lifespan, not for organs or systems within your body. Or try to figure out what are the environmental risk factors for childhood asthma, and what you can do to minimize your children’s chances of getting the disease [4-8]. If you get confused by what you find out, may I remind you that this problem is at least decades old; i.e. following epidemiology’s remarkable successes of the 1950s and 1960s in uncovering major risk factors for chronic diseases such as smoking, obesity, and high blood pressure [9,10].

This situation is threatening to erode the public trust in scientific research [11,12], but on a more fundamental level it casts serious doubts about the ability of epidemiology as a science to dissect complex interactions of low-magnitude risks [13]. Epidemiologists’ traditional response to these challenges has focused on calling for more refined methods, quality control, reliance on objective measures, and better statistical models to deal with measurement errors and uncertainty around models’ predictions [8,13-15]. While these are steps in the right direction for the science in general, I argue that they are of limited value for the study of complex diseases and lifestyle risk factors for reasons beyond the known limitations of observational epidemiology [16]. I base this opinion on two main factors; 1- our inherent inability to measure simultaneously and accurately multiple attributes of human behavior/exposures, and 2- the unpredictability of the role of individual components within complex interactions affecting population’s health. Below, I will discuss these briefly.

## Discussion

Most of the risk factors relevant to the study of complex diseases are lifestyle-related behaviors and exposures that require long time to produce small effects on disease’s risk. The smaller the risk and the more complex the causal universe, the more likely that measurement errors can lead to unpredictable and subsequently inconsistent results [8,13]. This situation applies to many chronic diseases, such as cancer, cardiovascular, psychiatric and neurodegenerative disease, for which the search for new lifestyle risk factors is a main focus of epidemiological research. As such, in order to produce robust predictions of these ailments, we need to measure precisely multiple behaviors/exposures over a long period of time (years, even decades). Apart from the technical futility of even trying to achieve this, the intensive probing resulting from such examination, will likely influence those under-investigation leading to biased estimates.

The previous point can be illustrated using one of the recent innovative approaches to measure lifestyle-related behaviors and exposures known as Ecological Momentary Assessment (EMA) [17]. Briefly, EMA is a framework that attempts to overcome the inadequacy of self-report, by providing real-time monitoring of behaviors/exposures [17]. Several technological advances have been adapted recently (phone apps, personal monitors, and biosensors) for real time recording of physical activity, food intake, stressors, drugs, or environmental exposures [18]. Such means undoubtedly have the potential to correlate better with important risks implicated in complex diseases than the crude recall techniques traditionally used (e.g. questionnaires). However, it is well recognized that EMA studies, even those that measure limited sets of behaviors, often place more burden on participants compared to more traditional means of assessment. This is because they generally require participants to periodically interrupt their daily activities to complete the measures [19]. The influence of assessment itself on the behavior assessed has been long recognized (reactive self-monitoring), even for the monitoring of simple behaviors [20,21]. As such, applying EMA to a multitude of behaviors/exposures involved in complex disease epidemiology will likely involve a substantial disruption of daily routine of participants. In the long term, compliance with demanding study procedures will likely decline, bringing the participants back to their usual routine at the expense of maintaining the fidelity of study protocol. In essence, the more the study participants abide by the study protocol, the less they become representative of similar individuals outside of the study sample (population), and vice versa.

Imagine an investigator wanting to measure accurately common factors implicated in the etiology of chronic diseases such as food intake, physical activity, and stress. As these attributes are repetitive, frequent, and diverse in nature and range, their continuous monitoring over a long period even using the most advanced gadgets will likely involve substantial intrusion on the daily routine of study participants (e.g. instrument charging, calibration, carrying, data input/download/export, etc.). Ease of measurement and perceived importance moreover, may lead study participants to differentially follow the study protocol for different attributes resulting in variable data quality. Incorporating physiological/biological assessments, or complementing monitoring with observations have been proposed to help deal with such limitations [17]. However, many of the behaviors invoked in complex disease epidemiology such as dietary habits and physical activity, do not have valid biological markers. Even if such markers do exist, the long period of monitoring required to study lifestyle risks of chronic disease will likely influence their assessment in a variety of ways including, natural development of tests and reagents, changing of quality standards, interactions due to acute and chronic conditions facing study subjects, changes due to aging (e.g. decline in kidney and liver functions), and finally market forces that can drive certain providers of testing materials out of business.

The second factor underlying my doubt about the ability of epidemiology to further the study of complex disease relates to the unpredictability of the role of individual components within complex interactions affecting population’s health. Much of the epidemiology of complex disease is built around a probabilistic causal paradigm, where the presence of a risk factor predicts an increase in the probability of the disease outcome. Accordingly, models commonly used in the epidemiology of chronic disease tend to produce fixed and one directional risk estimates. In real life however, this one-directional relationship is rarely the case, as complex interactions and feedback loops prevail [2]. Low physical activity leads to obesity, while obesity will limit physical activity, and such dynamic interaction cannot be sorted out based on the dichotomous causal/reverse-causal simplification. Population health from this perspective, represents a complex system, defined as “a collection of individual agents with freedom to act in ways that are not always totally predictable, and whose actions are interconnected so that one agent’s actions changes the context for other agents” [22]. Such systems are characterized by being non-linear with feedback loops, which allows for self-organization, and for small changes to have large effects that cannot be understood by looking at individual components [22-25]. For example, the collapse of inner city communities, with associated health burdens can be driven by a decision to reduce fire services, and subsequently can be prevented by modest interventions [23]. Applying predictive research models to such situations would have likely identified several proxies (e.g. poverty, housing conditions, insurance, access to care, social deprivation), and have made hence complex, and mostly unrealistic recommendations for interventions. Accordingly, we need a paradigm shift to address the rich and dynamic interactions between humans and their ever changing environments.

One of the main spheres that illustrate the inadequacy of our current epidemiological methods and concepts to our changing environments is the “online world”. Increasingly we became more connected, but perhaps more isolated, which is changing the notion of population, community, and peers beyond the traditional definitions used in health research. Much of religion, politics, and culture are taking place online nowadays and they are shaping a new sense of community and identity that defies classical geopolitical boundaries and have profound effect on people’s lifestyle and health choices. While the complexity of such systems can be a deterrent for researchers, what looks complex and unpredictable at one level, can be simple and predictable at another. For example, it can be hard to predict day-to-day climate or market behavior, while things become rather simple on a larger scale, such as seasons and economic cycles [22]. Picking the right level/scale therefore, can reveal much more about factors influencing complex diseases than applying multi-level frameworks currently trendy in epidemiology [8].

## Summary

It seems that epidemiology has reached its boundaries for what it can achieve in terms of complex diseases and their lifestyle risk factors, and that more studies with better design or measurement will unlikely lead to breakthroughs that can justify their cost. For such breakthroughs to happen, new approaches and paradigms, such as complexity theory, need to be adapted to the study of human health. Within complex systems, context and understanding the interactions between system components can be more important than quantifying the contribution of each component to disease risk. Thus, multi-disciplinary approaches involving social sciences can be beneficial in providing context information about lifestyle risk factors, while studies connecting disease trends to broader political-economic drivers influencing human activity, nutrition, and exposures, can provide pathways for solutions within the built, work, and recreational environments [26]. Within such framework, we need perhaps to start talking about ecological plausibility rather than biological plausibility, in recognition of interconnectedness between parts of complex systems beyond the sum of those parts. The challenges of measurement and complexity of human life for the epidemiology of complex disease will unlikely be resolved from within, but require new paradigms and tools. I fully understand that I am advancing several complex issues without entertaining the depth and breadth of the literature about them. My aim here is modest; to stimulate a healthy debate about the science of epidemiology of complex diseases and lifestyle risk factors.
